# Draft Genome Sequence of *Sphingobium fuliginis* OMI, a Bacterium That Degrades Alkylphenols and Bisphenols

**DOI:** 10.1128/genomeA.01323-17

**Published:** 2017-11-22

**Authors:** Masashi Kuroda, Yuka Ogata, Tatsuya Yahara, Takashi Yokoyama, Hidehiro Ishizawa, Kazuki Takada, Daisuke Inoue, Kazunari Sei, Michihiko Ike

**Affiliations:** a Division of Sustainable Energy and Environmental Engineering, Graduate School of Engineering, Osaka University, Suita, Osaka, Japan; b Center for Material Cycles and Waste Management Research, National Institute for Environmental Studies, Tsukuba, Ibaraki, Japan; c Department of Health Science, Kitasato University, Sagamihara-Minami, Kanagawa, Japan

## Abstract

*Sphingobium fuliginis* OMI is a bacterium that can degrade a variety of recalcitrant alkylphenols and bisphenols. This study reports the draft genome sequence of *S. fuliginis* OMI.

## GENOME ANNOUNCEMENT

Alkylphenols (APs) and bisphenols (BPs) are industrially important base materials used to produce surfactants and resins such as AP ethoxylates, phenol formaldehyde resins, epoxy resins, and polycarbonate resins. APs and BPs are also known as aquatic pollutants that are highly persistent, cause acute and chronic toxicities, and show estrogenic activity, threatening humans and aquatic ecosystems. *Sphingobium fuliginis* OMI was isolated from the rhizosphere of a giant duckweed, *Spirodela polyrhiza*, as a 4-*tert*-butylphenol-utilizing bacterium (1). To date, *Sphingobium fuliginis* strains OMI and TIK-1 are the only bacteria that have been reported as being capable of utilizing 4-*tert*-butylphenol as a sole carbon and energy source (1, 2). In addition to 4-*tert*-butylphenol, *Sphingobium fuliginis* OMI can degrade a wide variety of APs and BPs via a *meta*-cleavage pathway (3). The molecular mechanism underlying this degradation ability is of great research interest.

The genomic DNA of *Sphingobium fuliginis* OMI was extracted using an illustra bacteria genomicPrep Mini Spin Kit (GE Healthcare Japan, Tokyo, Japan). Sequencing was performed on a Roche 454 genome sequencer FLX. Using a Celera assembler, version 5.3, reads of 179,321,730 bp were assembled into a draft genome sequence of 113 contigs with a G+C content of 64.5% for a total of 5,532,952 bp, an *N*_50_ of 152,384 bp, and a maximum contig size of 383,981 bp. A total of 5,315 coding sequences (CDSs) were predicted by the Rapid Annotations using Subsystems Technology (RAST) prokaryotic genome annotation server (4).

The genome includes seven genes that encode extradiol dioxygenase, which is a key enzyme in the *meta*-cleavage pathway of APs and BPs. Genes related to the pathway from 2-hydroxymuconate semialdehyde to acetyl coenzyme A were also present, which is consistent with the fact that strain OMI can grow on 4-*tert*-butylphenol. Genes related to chemotactic responses and flagellar assembly (*che*, *fli*, *mot*, and *flg*) might underlie chemotaxis toward the root zone of *Spirodela polyrhiza*, an important trait of rhizobacteria (5). This is the first report on the draft genome sequence of *Sphingobium fuliginis*.

### Accession number(s).

The annotated draft genome sequence of *Sphingobium fuliginis* OMI was deposited at DDBJ/EMBL/NCBI under accession no. BEWI01000001 to BEWI01000034.
